# Comparisons between the Dosimetric and Clinical Outcomes of Tomotherapy and 3D Conformal Radiotherapy in Gastric Cancer Treatment

**DOI:** 10.31557/APJCP.2019.20.2.595

**Published:** 2019

**Authors:** E Kucuktulu, A F Yurekli, M Topbas, C Kece, A Guner, U Kucuktulu

**Affiliations:** 1 *Consultant Oncologist, University of Health Sciences, Kanuni Research and Training Hospital, Dept of Radiation Oncology, *; 3 *Professor of Public Health, Karadeniz Technical University, Medical School, Department of Public Health,*; 5 *Associate Professor of Surgery, Karadeniz Technical University, Medical School,*; 6 *Professor of Surgery, University of Health Sciences, Kanuni Research and Training Hospital Department of General Surgery, Trabzon,*; 2 *Medical Physicist MSc, Yildirim Beyazit University, Medical School, Ataturk Research and Training Hospital, Department of Radiation Oncology, Ankara,*; 4 *Associate Professor of Surgery, Bahcesehir University, Medical School, Department of General Surgery, Istanbul, Turkey.*

**Keywords:** Gastric cancer, tomotherapy, 3D conformal radiotherapy

## Abstract

**Introduction:**

Previous studies comparing tomotherapy (TOMO) and three dimensional (3D) conformal radiotherapy (3DCRT) in gastric radiotherapy are limited and tend to be based on dosimetry. The aim of the present study was to evaluate the clinical outcomes of these two treatment modalities.

**Methods:**

A total of 51 patients diagnosed with gastric cancer who were treated with postoperative adjuvant chemoradiotherapy and had subtotal/total gastrectomy and D2 lymphatic dissection were recruited to the present study: 30 patients were treated with TOMO and 21 patients were treated with 3DCRT.

**Results:**

The 3DCRT and TOMO treatment regimens were compared. There was no difference in planning target volume (PTV) 95%, but TOMO was statistically significant in regard to PTV 105% (P<0.05). TOMO was also significantly different when compared with 3DCRT when evaluating liver mean dose, liver V40, right/left kidneys mean dose, right/left kidneys V20 and spinal cord mean dose values (P<0.05). Grade 2 acute side effects were more frequent (85.7%) following 3DCRT. In addition, the median overall survival time for TOMO treated patients was 62 months while in 3DCRT treated patients it was 22.05 months. The difference in disease free survival was also significantly increased in patients treated with TOMO (66.7% vs. 19.0%; P<0.05).

**Conclusion:**

TOMO treatment resulted in lower acute side effects with better patient survival following gastric cancer radiotherapy.

## Introduction

Chemoradiotherapy is the standard strategy employed for the management of gastric tumors as it can target through the gastric wall and tumor positive nodes (Macdonald et al., 2001). The SWOG 9008/INT0116 study demonstrated sustained benefits in overall survival (OS) and relapse free survival (RFS) in the ten year follow up (Smalley et al., 2012). 

**Table 1:** 51 Gastric Cancer Patients Who were Stage IB/IIIC and had Subtotal/Total Gastrectomy and D2 Lymphatic Dissection

	3DCRT n (%)	TOMO n (%)
Median	55	58
Surgery type		
Subtotal gastrectomy	7 (33.3)	15 (50.0)
Total gastrectomy	14 (66.6)	15 (50.0)
Stage (AJCC 7th)		
IB	1 (4.8)	2 (6.7)
IIA	3 (14.3)	3 (10.0)
IIB	3 (14.3)	3 (10.0)
IIIA	4 (19.0)	2 (6.7)
IIIB	7 (33.3)	9 (30.0)
IIIC	3 (14.3)	11 (36.7)
Histology		
Adenocarcinoma	11 (52.4)	23 (76.7)
Signet ring cell	10 (47.6)	6 (20.0)
Other	0 (0)	1 (3,3)

3DCRT, 3 Dimensional conformal radiotherapy; TOMO, Tomotherapy; AJCC, American joint committee of cancer.

**Table 2:** Comparing OAR Doses of TOMO and 3DCRT Treatments

	Tomotherapy		3DCRT		P
	Mean	Std. Deviation	Mean	Std. Deviation	
PTV 95%	99.73	0.64	99.62	0.74	0.534*
PTV 105%	0.68	0.98	37.14	10.91	<0.001*
Liver Mean	15.98	3.76	22.35	6.43	<0.001**
Liver >V40	6.83	3.01	19.52	5.88	<0.001*
R-Kidney Mean	6.54	2.71	10.71	3.08	<0.001**
R-Kidney >V20	3.4	3.82	15.26	6.26	<0.001*
L-Kidney Mean	7.9	2.36	17.03	6.27	<0.001**
L-Kidney >V20	7.53	5.09	30.67	15.28	<0.001*
Spinal Cord Mean	11.97	5.07	30.15	9.75	<0.001**

*, Mann Whitney U test; **, Student t test; 3DCRT, 3 Dimentional conformal radiotherapy; TOMO, Tomotherapy.

**Table 3:** Comparing Status of Patients, Stages, Acute and Late Effects of TOMO and 3DCRT Treatments

	Tomotherapy		3DCRT		
	n	%	n	%	P
Stage					
IB	2	6.7	1	4.8	*
IIA	3	10	3	14.3	
IIB	3	10	3	14.3	
IIIA	2	6.7	4	19	
IIIB	9	30	7	33.3	
IIIC	11	36.7	3	14.3	
Status					
Live	20	66.7	4	19	0.001
Ex	10	33.3	17	81	
Acut side effect					
No	25	83.3	0	0	<0.001
Yes	5	16.7	21	100	
Late side effect					
No	26	86.7	17	81	0.702**
Yes	4	13.3	4	19	
Surgical type					
1	15	50	7	33.3	0.371
2	15	50	14	66.7	

*, invalid chi-square value; **, Fisher’s exact test.

**Table 4:** Acute and Late Side Effects

		B	SE	Wald	p	OR	95.0% CI for Exp(B)	
							Lower	Upper
Step 1	Late Side Effect	1.45	0.64	5.1	0.024	4.25	1.21	14.9
	Tomo vs 3DCRT	3.86	1.37	7.9	0.005	47.61	3.22	704.09
	Acute Side Effect	-0.04	0.67	0	0.955	0.96	0.26	3.57
	Surgical type	0.59	0.52	1.29	0.256	1.8	0.65	4.96
Step 2	Late Side Effect	1.45	0.64	5.22	0.022	4.27	1.23	14.82
	Tomo vs 3DCRT	3.9	1.23	10.07	0.002	49.3	4.44	547.41
	Surgical type	0.58	0.51	1.3	0.254	1.79	0.66	4.87
Step 3	Late Side Effect	1.27	0.6	4.52	0.034	3.56	1.1	11.5
	Tomo vs 3DCRT	3.48	1.13	9.46	0.002	32.41	3.53	297.42

Backward LR Cox Regression Analysis

**Figure 1:**
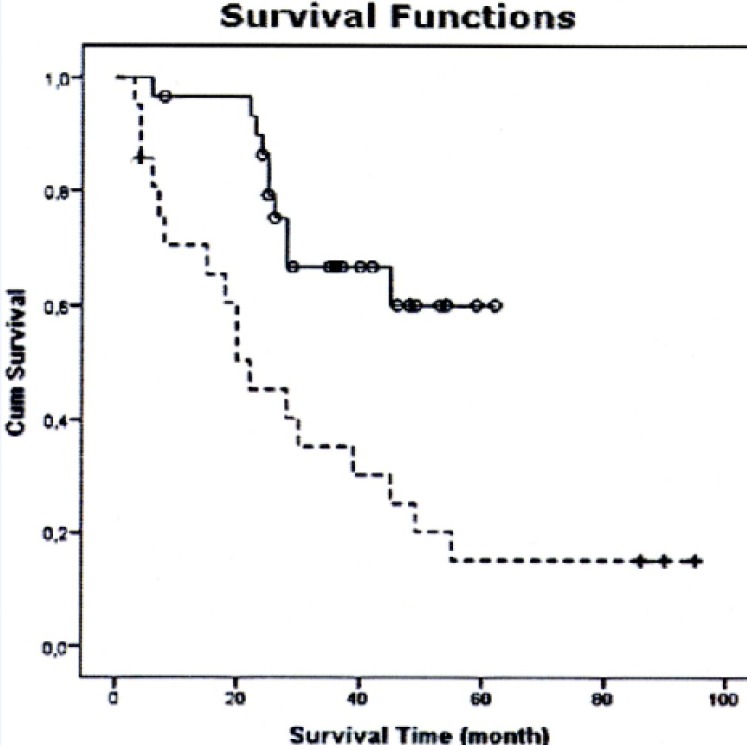
The Median Overall Survival Time for TOMO Treated Patients and 3DCRT Treated Patients

Initially, the use of two opposing fields was preferred in radiotherapy. However, the two large parallel opposed radiation fields, which cover both the tumor bed and regional lymph nodes, has led to poor treatment compliance: only 65% of patients complete the planned chemoradiotherapy regimen (Macdonald et al., 2001). In earlier studies of three-dimensional conformal radiotherapy (3DCRT), grade III acute gastrointestinal (GI) toxicity was observed in >50% of cases (Kassam et al., 2006). Multi-field techniques have subsequently gained popularity over time (Allal et al., 2005). Image Guided Intensity Modulated Radiotherapy (IMRT) enabled the preferential sparing of adjacent organs at risk and reduced potential toxicity (Dahele et al., 2010). Yovino et al., (2011) reported that using IMRT in chemoradiation treatments resulted in significantly less toxicity in the upper and lower gastrointestinal system when compared with 3DCRT. The best target volume coverage and sparing of liver and kidneys was observed in the IMRT regimen when compared with conventional techniques. Clinical studies comparing the outcomes of 3DCRT and IMRT are relatively few (Milano et al., 2006; Minn et al., 2010; Liu et al., 2014; Chopra et al., 2015). Some of these studies reported similar results for 3DCRT and IMRT in regard to toxicity and local control (Chopra et al., 2015). Wang et al., (2017) compared the dosimetric parameters of IMRT, volumetric-modulated arc therapy and tomotherapy (TOMO) in the adjuvant treatment of gastric cancer. In this study it was reported that TOMO provided better dose conformity and homogeneity and dose sparing of the bowel, bone marrow, kidneys and liver.

In the present study, the effects of TOMO on patient survival were evaluated as TOMO has been observed to provide better target volume coverage, thereby enabling superior local control. The present study also retrospectively reports that TOMO decreased the occurrence of side effects, thereby reducing the number of treatment gaps required in chemotherapy and radiotherapy regimes for gastric cancer treatment.

## Materials and Methods

*Patients and methods*

Between July 2009 and July 2016, patients diagnosed with gastric cancer were treated at Kanuni Training and Research Hospital, I. Lale Atahan Radiation Oncology Clinic (Trabzon, Turkey) with regimens of postoperative adjuvant chemoradiotherapy. The present study retrospectively evaluated 51 gastric cancer patients who were stage IB/IIIC and had subtotal/total gastrectomy and D2 lymphatic dissection (Table 1). Stage IB (T2N0) patients (n=3) received radiotherapy as tumor proximity was close to the surgical margins.

In all patients, a total dose of 4,500 cGy in 180 cGy fractions of radiotherapy was administered 5 days a week for 5 weeks. On the first and last 4 days of radiotherapy treatment, 5-Floutouracil (5-FU; 425 mg/m^2^) and Folinic acid (20 mg/m^2^) intravenous bolus were given to all patients. 

Once written informed consent was obtained, patients were placed in a supine position with wing board immobilization. All computed tomography (CT) evaluations were carried out using a Biograph True Point PET and Siemens CT Simulator with a 3 mm slice thickness. In all patients, CT scans were performed between the 6th cervical vertebra and the 5th lumbar vertebra.

The delineation of the clinical target volume (CTV), developed by Tepper and Gunderson, was based on the location and extent of the primary tumor (T category) and the location and extent of known nodal involvement (N category) (Tepper and Gunderson, 2002).

In 30 patients, TOMO was employed using Hi-Art Tomotherapy and in 21 patients the 3DCRT technique was applied using the Electa Synergy Platform Linear Accelerator. These 2 groups were compared for the following parameters: Liver mean dose, liver percentage of the volume receiving a dose of > 40 Gy, right/left kidneys mean dose, right/left kidneys percentage of the volume receiving a dose of > 20 Gy, spinal cord mean dose, planning target volume (PTV) 95% and PTV 105%.

3DCRT was carried out using the Precise Plan Release 2.16-28.76 treatment planning system; a 3 field planning technique was applied. In order to keep kidney dose at a minimum, optimal values were given to the collimator: 1 anterior and 2 lateral fields at the liver site were used. The photon beam doses were calculated using the pencil-beam algorithm.

Three major factors were taken into consideration for the TOMO planning system: Field width, pitch and modulation factor. In the present study, the field width, pitch and modulation factor were 2.5 cm, 0.287 and 2.2, respectively. 

As described in the International Commission on Radiation Units and Measurements 50 and 62 reports, in each treatment plan the present study evaluated the PTV 95% (42.75 Gy) and 105% (47.25 Gy) prescribed doses.

The radiotherapies of the patients in this study have been completed between June 2009 to April 2016. In this time period there was no upgrade to the radiotherapy systems except software upgrades. We treated all patients in the same center with identical treatment protocols.

The acute and late side effects during therapy and the follow-up period were evaluated with Common Toxicity Criteria Version 4.

All statistical analyses was performed using SPSS (SPSS, Inc., Chicago, IL, USA; licensed for Karadeniz Technical University). For statistical analysis, the Shapiro-Wilk, Mann-Whitney U, Student’s t-test and Chi-squared tests were used. P<0.05 were considered to indicate a statistically significant difference. Cox regression analyses were carried out for determining the factors effective on prognosis.

## Results

When the 3DCRT and TOMO treatment regimens were compared, there was no significant difference in PTV 95%. However, TOMO was significantly lower for PTV 105% values (P<0.05); PTV 105% values were higher in those treated with 3DCRT (mean, 37.14).

When comparing the liver mean dose, liver percentage of the volume receiving a dose of >40 Gy, right/left kidneys mean dose, right/left kidneys percentage of the volume receiving a dose of >20 Gy and spinal cord mean dose values, TOMO and 3DCRT treatments were significantly different, with TOMO producing the most beneficial results (P<0.05; Table 2).

When evaluating the patients according to Common Toxicity Criteria Version 4, grade 2 acute side effects were more frequent (85.7%) in patients treated with 3DCRT. While grade 3 side effects were not observed in those treated with TOMO; however, they were identified in 9.5% of 3DCRT treated patients. In addition, late side effects were observed in 13.3% and 19.0% of patients treated with TOMO and 3DCRT, respectively; there was no significant differences in the incidence of late side effects (P>0.05; Table 3).

The median overall survival time for TOMO treated patients was 62 months while in 3DCRT treated patients it was 22.05 months. The difference in disease free survival was significantly increased in patients treated with TOMO (66.7% vs. 19.0%; P<0.05; Figure 1) (Table 4).

## Discussion

In TOMO, a type of IMRT technique, radiation delivery is provided by a rotating gantry around the patient and is most commonly used in gastric cancer radiotherapy. TOMO provides superior dose conformity and homogeneity and dose sparing of the liver and kidneys (Wang et al., 2017). Although dosimetric studies comparing the clinical effectiveness of IMRT and 3DCRT have been reported previously, there have not been any clinical studies demonstrating the benefits of using the TOMO treatment system over 3DCRT and IMRT.

The two large parallel-opposed radiation fields technique has been reported to be associated with severe hematologic and GI toxicities. The incidences of grade 3 or higher hematologic and GI toxicities have been reported to be 54% and 33%, respectively (Macdonald et al., 2001). The high incidences of acute GI and hematologic adverse events often result in gaps between or missed chemotherapy treatments, which in turn negatively affect patient prognosis (Wang et al., 2017). 

The radiation dose distribution can be improved in targeted areas while reducing the radiation dose in normal organs in IMRT (Ringash et al., 2005). Although IMRT provides superior treatment tolerance and reduces the risk of damage to healthy tissues, its advantageous effects on survival have not been reported in the literature (Hawrylewicz et al., 2016). In the present study, although the number of patients in stage IIIC were higher in the TOMO treated group, the survival rate was significantly better in those treated with TOMO. In addition, the disease free survival in the TOMO treated group was 66.7%. Late side effects were only observed in 13.3% of TOMO treated patients; the side effects documented were classed as grade 1 and therefore were not life threatening, nor did they affect patient performance. 

A total of 83.3% of TOMO treated patients did not develop acute side effects; however, all of the 3DCRT treated patients developed acute side effects. Zue et al., (2012) reported that in gastric cancer patients treated with IMRT the grade I-II side effects were much more prominent (nausea 36.0%, vomiting 21.5% and diarrhea 12.9%). Minn et al., (2010) also reported that the incidence of toxicities were similar following IMRT and 3DCRT treatments (61.2% vs. 61.5%). In the present study, when compared with these results the toxicities observed following TOMO treatment were quite low. Grade 2 diarrhea was observed in 85.7% of 3DCRT patients, while it was reported in only 16.7% of the TOMO treated patients. When new contouring techniques are used, the treatment field importantly diminishes. TOMO plans provide better results for receiving 95% of doses in CTV. In 3D CRT, larger safety margins are required to reach 95% coverage which results more small intestines to be included in treatment field that consequently causes more side effects. This is notable as 5-FU was used concomitantly in the protocol and diarrhea was observed with a very low incidence. Thus, to the best of our knowledge, this is the first clinical study reporting that TOMO patients develop fewer side effects.

A previous study reported that TOMO achieved a better dose homogeneity for PTV coverage than IMRT (Wieland et al., 2004). TOMO technology can also be used to spare more liver and kidney volume, which may be beneficial for patients (Wang et al., 2017). In the present study, TOMO treatment produced a significantly lower liver mean dose, liver percentage of the volume receiving a dose of >40 Gy, right/left kidneys mean dose, and right/left kidneys percentage of the volume receiving a dose of >20 Gy. These results are in accordance with those of previous dosimetric studies. In addition, the results were reflected in the clinical outcomes in the present study as patients treated with TOMO completed the treatment without treatment breaks as their performance conditions were greater than those treated with IMRT. Since tomotherapy resulted in less side effects, it caused less gaps or missed chemotherapy treatment which affected patients overall survival.

When evaluating the histological results, the number of signet cell type side group in stage IIIB 3DCRT treated patients was greater (71.4% vs. 22.2%). In addition, the stage IIIC subgroup of patients was greater in the 3DCRT treatment group: 66.7% and 27.3% in the 3DCRT and TOMO groups, respectively. When all stages were evaluated, signet ring cell histology was observed in 47.6% of patients treated with 3DCRT compared with 20.0% of patients treated with TOMO. In the retrospectively evaluated patient group, there was more signet cell histology in the 3DCRT treated group. In total, 36.7% of TOMO treated patients were stage IIIC patients, whereas in the 3DCRT treatment group, stage IIIC patients formed only 14.3% of the total number of patients. Therefore, the advantageous effect of TOMO on patient survival was independent of histological type; except from this parameter, all other parameters were observed in parallel in both groups.

TOMO is currently one of the most sophisticated forms of IMRT implemented in clinical practice. As it is a relatively new technique, the effects on survival and local control, as well as the long term toxicities have not been fully elucidated (Ramsey et al., 2007). The current advances in radiotherapy technologies and further improvements in treatment outcomes have reduced the number of early and late radiotherapy toxicities, however, this still remains a challenge. The present study, although retrospective and with a limited number of patients, to the best of our knowledge is one of the first studies reporting clinical results of TOMO treatment in gastric cancers. This study includes the patients with long follow-up. We excluded the patients with shorter follow-up period. When relevant follow-up period (at least 5 years) is reached for remaining patients we are planning to re-evaluate the results. It is quite difficult to find stage III C gastric cancer patients with 5 years follow-up further clinical studies that recruit a higher number of patients will be highly beneficial. In conclusion, TOMO may provide better dose distribution for targets with longer and more complex shapes such as in gastric cancer treatment and thus, may produce better clinical results. From 2016 on all gastric cancer patients received tomotherapy in our radiotherapy center after we realized less side effects tomotherapy caused. 

## Conflict of interest

The authors declare that there is no conflict of interest.
